# The effect of Quorum sensing inhibitors on the evolution of CRISPR-based phage immunity in *Pseudomonas aeruginosa*

**DOI:** 10.1038/s41396-021-00946-6

**Published:** 2021-03-10

**Authors:** Jenny M. Broniewski, Matthew A. W. Chisnall, Nina Molin Høyland-Kroghsbo, Angus Buckling, Edze R. Westra

**Affiliations:** 1 Biosciences, Environment and Sustainability Institute, University of Exeter, Penryn, UK; 2 Department of Plant and Environmental Sciences, University of Copenhagen, Frederiksberg, Denmark

**Keywords:** Microbiology, Antimicrobials

## Abstract

Quorum sensing controls the expression of a wide range of important traits in the opportunistic pathogen *Pseudomonas aeruginosa*, including the expression of virulence genes and its CRISPR-*cas* immune system, which protects from bacteriophage (phage) infection. This finding has led to the speculation that synthetic quorum sensing inhibitors could be used to limit the evolution of CRISPR immunity during phage therapy. Here we use experimental evolution to explore if and how a quorum sensing inhibitor influences the population and evolutionary dynamics of *P. aeruginosa* upon phage DMS3vir infection. We find that chemical inhibition of quorum sensing decreases phage adsorption rates due to downregulation of the Type IV pilus, which causes delayed lysis of bacterial cultures and favours the evolution of CRISPR immunity. Our data therefore suggest that inhibiting quorum sensing may reduce rather than improve the therapeutic efficacy of pilus-specific phage, and this is likely a general feature when phage receptors are positively regulated by quorum sensing.

## Introduction

The increase in antimicrobial resistance has led to a resurgence of interest in phage therapy, where bacterial viruses (phage) are used to treat bacterial infections [1–4]. *Pseudomonas aeruginosa* is an important source of nosocomial infections and potential target for phage therapy [5]. In the laboratory, bacteria tend to rapidly evolve resistance to phage [6], which in a clinical setting might limit the efficacy of the therapeutic phage application [2]. Rapid phage resistance evolution is often due to cell surface alterations that interfere with phage adsorption, or the acquisition of CRISPR-based immunity, where bacteria insert phage-derived sequences into CRISPR loci on the host genome, which are used to detect and destroy the same or closely related phage during future infections [7–10]. In the case of *P. aeruginosa* PA14, both these mechanisms of phage resistance have been shown to evolve at high frequencies in the laboratory, but the dominant mechanism depends on environmental variables, such as the microbial community context in which infections occur [11], the level of phage genetic variation [12], and the force of infection [13, 14]. The force of infection is an important determinant of CRISPR immunity evolution because unlike surface-based resistance, which carries a fixed fitness cost, CRISPR immunity is associated with an infection-induced fitness cost due to the expression of phage genes prior to clearance of the infection [14, 15]. Hence, in environments with a high force of infection, surface-based resistance is favoured over CRISPR immunity and vice versa. Because evolution of surface-based resistance, but not CRISPR immunity, is associated with virulence trade-offs [11], being able to manipulate which type of phage resistance evolves and limiting overall resistance development could have important clinical impacts.

It was recently demonstrated that Quorum Sensing (QS) controls expression of the CRISPR-*cas* immune system in *P. aeruginosa* [16] and in *Serratia* [17]. Based on these findings it was suggested that chemical QS inhibitors could be leveraged to limit the evolution of CRISPR-based immunity in a clinical setting [16]. QS is a well-studied bacterial communication system which allows bacteria to monitor local cell densities based on the concentration of small autoinducer molecules (AIs). *P. aeruginosa* PA14 encodes several QS systems, including two that use acyl homoserine lactone (AHL) signalling (LasIR and RhlIR), and the PQS system (for *Pseudomonas* quinolone signal) (reviewed in [18]). The AHL-based QS systems are involved in regulation of around 6% of the genes encoded by *P. aeruginosa* [19–21], including CRISPR-*cas* [16] as well as genes involved in virulence and biofilm formation [22]. The LasIR and RhlIR systems are organised in a hierarchical manner with LasIR at the top of the hierarchy [23, 24]. LasI produces the AHL signal molecule 3-oxo-C12-homoserine lactone (3OC12-HSL), which is released from the cell from where it diffuses until it is taken up by the same or other bacteria in the environment. As cell densities increase, the concentration of the 3OC12-HSL autoinducer within cells increase, which is sensed through the LasR receptor. Beyond a threshold in the 3OC12-HSL concentration, LasR-3OC12-HSL complexes activate target genes, including both LasI and RhlIR. RhlI produces the AHL signal molecule C4-homoserine lactone (C4-HSL). This autoinducer is detected by the RhlR receptor, leading to activation of a second wave of QS genes [25, 26] (reviewed in [27, 28]).

Because LasIR and RhlIR QS systems control such a broad range of genes, it remains difficult to predict how QS inhibitors impact phage therapy. For example, these QS systems of *P. aeruginosa* activate not only expression of the CRISPR-*cas* immune system [16] but also that of its Type IV pili [29], which serve as receptors for many *Pseudomonas* phage [29–34]. Manipulation of QS may therefore affect both the resistance levels of the host and the infectivity levels of the phage that require type IV pili for infection. Since it remains unclear what the overall effect of QS inhibitors is on the efficacy of phage therapy, we measured how chemical inhibition of QS using Baicalein [35] impacts the ecological and evolutionary interaction between *P. aeruginosa* PA14 and its pilus-specific phage DMS3vir under in vitro laboratory conditions. Our data show that QS inhibition triggers phenotypic changes in the bacterial population that result in partial immunity to phage due to reduced adsorption to the host cells, and the resulting decrease in phage infection frequencies favours the evolution of CRISPR immunity over surface-based resistance. These data suggest that inhibition of QS can reduce the efficacy of phage therapy in a clinical context when QS inhibition results in reduced expression of the phage receptor resulting in less frequent successful infections.

## Methods

### Bacterial strains and phage

We used *P. aeruginosa* UCBPP-PA14 (referred to as WT), *P. aeruginosa* UCBPP-PA14 *cas7::LacZ* (referred to as CRISPR-KO, since it carries a non-functional CRISPR-*cas* system) [36], a previously described CRISPR-KO-derived surface mutant (referred to as sm) [14], and *P. aeruginosa Tn::pilA* (referred to as *pilA* KO) [37], a CRISPR-*cas* immune isolate of *P. aeruginosa* UCBPP-PA14 possessing two spacers targeting the phage DMS3vir (referred to as BIM2 – Bacteriophage insensitive Mutant, previously described in [14]), as well as a double synthase mutant strain of *P. aeruginosa* UCBPP-PA14, Δ*lasI* Δ*rhlI*, (referred to as QS mutant) [16]. Cultures were started from a stock stored at −80 ^o^C and were grown in M9 media at 37 ^o^C for 3 days with transfers to fresh media each day to allow acclimatisation. For all infection experiments we used the previously described mu-like phage, DMS3vir [36]. We also used DMS3*vir-acrIF1* described in [38], which carries the anti-CRISPR gene *acrIF1* that effectively blocks the CRISPR-*cas* immune systems of *P. aeruginosa* PA14 [38–40] during streak assays to determine the mechanistic basis of phage resistance. Full datasets for all experiments are available at: https://datadryad.org/stash/share/Tt20sh0vKBz6gkoqKWHkzkSYMUdFj0qMBEiq1tUdAoo.

### Evolution experiments

Infection experiments were carried out in glass vials containing 6 ml of M9 media with 0.2% glucose supplemented with 100 µM Baicalein (Cayman Chemical) dissolved in DMSO, and 10^6^ cfu/ml *P. aeruginosa* WT. The same experiment was carried out concurrently with the QS mutant in the presence or absence of 2 µM 3OC12-HSL and 10 µM C4-HSL (Sigma) dissolved in DMSO. Control treatments received the same amount of DMSO. Total amounts of 10^4^ plaque forming units (pfu) (low phage), 10^7^ pfu (mid phage) or 10^9^ pfu (high phage) were added at the same time as the bacteria. Microcosms were incubated at 37 °C while shaking at 180 rpm. Every 24 h microcosms were sampled and transferred to fresh media at a 1:100 dilution. Samples from the infected cultures were taken prior to each transfer and chloroform treated to kill bacteria as described previously [38]. 5 µl of a serial dilution of chloroform treated sample was spotted onto a lawn of sensitive bacteria (CRISPR-KO strain). Plaques were counted and densities were calculated as pfu/ml. Bacteria were sampled at 3 days post infection to analyse the resistance mechanisms that evolved in response to phage infection.

### Resistance mechanism analysis

To determine whether bacteria had evolved phage resistance, and whether this was surface-based resistance or CRISPR-based immunity, we performed cross-streak assays as described previously [14]. Briefly, bacterial colonies were isolated by plating on LB agar, 24 colonies per replicate were picked at random per replicate, grown overnight in M9 media supplemented with 0.2% glucose and then streaked across the ancestral phage (DMS3vir) and the phage carrying the anti-CRISPR gene (DMS3*vir-acrIF1*) [38]. Colonies which were resistant to the ancestral phage but not the phage carrying an anti-CRISPR gene were classed as CRISPR immune, whereas colonies that were resistant against both DMS3vir and DMS3*vir-acrIF1* were classed as having sm (surface mutation) resistance. Colonies that were not resistant to either phage were classed as sensitive. Results were confirmed with PCR of both of the CRISPR loci which *P. aeruginosa* possesses to check for spacer acquisition in WT and QS mutant clones as described previously [14].

### Phage adsorption

WT *P. aeruginosa* was grown for 24 h in media supplemented with either DMSO (control) or the QS inhibitor. Cultures were centrifuged at 3500 rpm to form a pellet. The supernatant was discarded and the pellets were resuspended in 5 ml of pre-heated (37 ^o^C) medium with or without the QS inhibitor to OD_600_ 0.2. Cultures were incubated at 37 ^o^C, 180 rpm for 3 h to achieve mid-log phase and then diluted with pre-heated growth medium to a final volume of 15 ml. 6 ml of each sample was taken and stored on ice to be used to quantify bacterial density at the start of the experiment, as described below. The remaining 9 ml was put back in the incubator at 37 ^o^C, 180 rpm. One tube of plain growth media was also processed as a control. Cultures were incubated for 5 min to allow for temperature equilibration. 1 ml of pre-warmed phage in growth media was added to each culture with a final concentration of 2 × 10^5^ pfu/ml. Cultures were mixed and a sample taken and added to Eppendorf vials containing 100 µl of chloroform, vortexed and stored on ice. This process was repeated at 3, 6, 9, 12, 15, 20, 25, 30, 40 and 50 min post infection. Phage densities were calculated by spotting virus samples isolated by chloroform extraction on a lawn of CRISPR KO bacteria. Bacterial densities were determined by plating dilutions of the original cultures, which were taken at the start of the experiment and stored on ice, on LB Agar plates and counting CFUs. The fraction of free phage particles at each time point were calculated relative to the phage titre at T0.

### Phage elimination assays

To measure CRISPR-mediated phage elimination over longer timespans, we mixed the Bacteriophage Insensitive Mutant BIM2, which possesses 2 spacers targeting DMS3vir in its’ CRISPR array with DMS3vir in glass microcosms containing 6 ml of M9 media supplemented with 0.2% glucose or DMSO with or without a QS inhibitor (100 µM Baicalein) or AIs (2 µM 3OC12-HSL + 10 µM C4-HSL) and inoculated with 10^6^ cfu/ml of the BIM. Cells were then infected with 10^6^ pfu/ml of phage DMS3vir. Vials were incubated at 37 °C while shaking at 180 rpm for 24 h and then sampled. Phage titres were determined via chloroform extraction and spot assay, as described above.

### Competition experiments

We measured fitness by competing the BIM2 targeting DMS3vir against either the phage-sensitive CRISPR-KO strain or against the phage-resistant surface mutant (the *pilA* KO or sm strain). A CRISPR immune strain with 2 phage-targeting spacers was used to avoid confounding effects from the evolution of “escape phage” that carry mutations in their target sequences [38, 41]. Competitions were performed in glass microcosms containing 6 ml of M9 media with 0.2% glucose, and containing either the inhibitor (100 µM Baicalein), the autoinducers (2 µM 3OC12-HSL + 10 µM C4-HSL), or both the QS inhibitor and inducers at these same concentrations. Controls contained the same volume of DMSO only. Microcosms were then inoculated with 10^6^ colony forming units (cfu)/ml of a 1:1 mix of the competing strains. Cells were infected with 10^6^ pfu/ml of phage DMS3vir. Vials were incubated at 37 °C while shaking at 180 rpm for 24 h and then sampled. The relative frequencies of each strain were determined via serial dilution and plating on 1.5% LB agar plates containing 50 mg/ml X-gal (5-bromo-4-chloro-3-indolyl-β-D-galactopyranoside). In each competition experiment one of the competing treatments carried the *LacZ* gene which produces the enzyme β-galactosidase, causing colonies to turn blue when grown on media containing X-gal. This allowed us to visually distinguish competing clones for quantification of their relative frequencies. Relative fitness was calculated as rel. fitness = [(fraction strain A at t = x) * (1 – (fraction stain A at t = 0))] / [(fraction strain A at t = 0) * (1 – (fraction strain A at t = x)]).

### Motility assays

*P. aeruginosa* WT or the QS mutant were streaked onto 1.5% LB agar plates and grown overnight at 37 ^o^C. In total, 6 ml M9 media was supplemented with 0.2% glucose and was inoculated with either the WT or QS mutant and grown for 24 h at 37 ^o^C, while shaking at 180 rpm. Swimming ability was determined by inserting a pipette tip containing WT bacteria or the QS mutant halfway through a 0.3% LB agar plate. Swarming assays were performed by spotting 5 µl of undiluted liquid culture onto a 0.5% LB agar plate. All motility assay plates were incubated for 24 h at 37 ^o^C. Motility was measured as the diameter of bacterial growth in mm at the widest point which intersected the point of inoculation.

## Results

### The effect of QS inhibition on phage resistance evolution

**Fig. 1:**
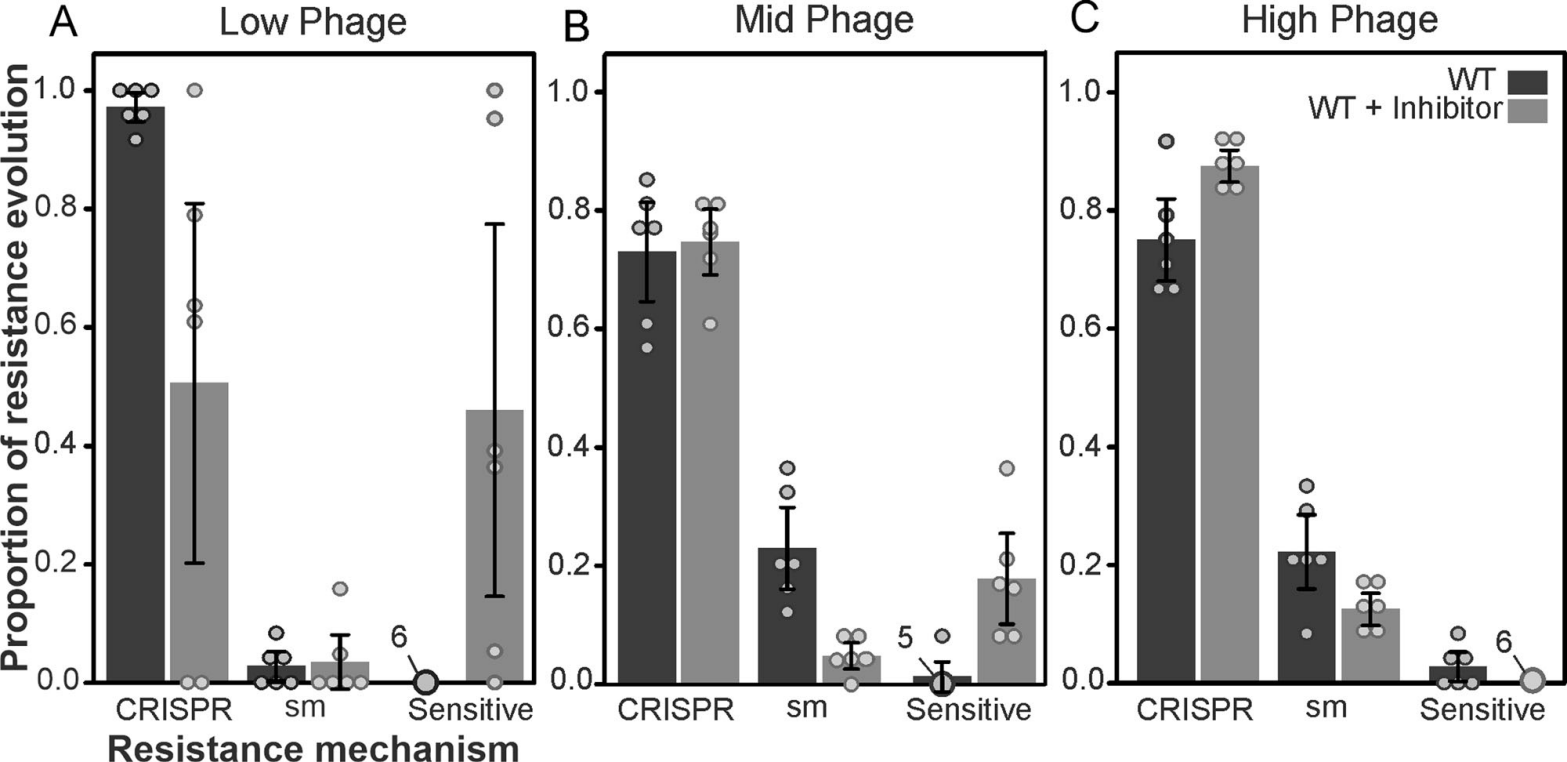
Inhibiting QS results in reduced phage sensitivity and prolonged phage persistence at low phage densities. Observed resistance mechanism evolution in *P. aeruginosa* PA14 WT or surface mutant (sm) supplemented with DMSO (dark grey bars/points) or 100 µM QS inhibitor Baicalein (light grey bars/points) and at 3 days post infection with (**A**) 10^4^ pfu (**B**) 10^7^ pfu (**C**) 10^9^ pfu of phage DMS3vir. Bars represent mean proportions of each of the different bacterial phenotypes (i.e., the proportion of bacteria in the population that evolved CRISPR immunity, acquired surface mutations or that remained sensitive), error bars indicate 95% confidence interval, points represent individual replicates, *N* = 6.

To measure whether chemical QS inhibitors can indeed be used to limit the evolution of CRISPR-based immunity against a pilus-specific phage, we exposed WT PA14 to either 10^4^, 10^7^ or 10^9^ pfu of phage DMS3vir in the presence or absence of Baicalein, a potent QS inhibitor [35] (Fig. 1).

In our control, (WT supplemented with DMSO) evolution of CRISPR-based immunity decreased (and surface-based resistance (sm) increased) with increasing phage titre, as reported previously [11, 13, 14], with almost all bacteria having evolved CRISPR immunity in the low phage treatment compared to around three quarters of the population in the mid and high phage treatments (Fig. 1). However, when QS inhibitors were added, we observed changes in the patterns of phage resistance evolution that were contrary to our expectation of a general reduction in CRISPR-mediated immunity. Specifically, in the presence of a QS inhibitor there was a significant increase in the persistence of phage-sensitive bacteria during infection with 10^4^ pfu (Mann–Whitney U = 3, *p* < 0.05) or 10^7^ pfu (U = 1, *p* < 0.01) of DMS3vir. Inhibition of QS was also associated with a reduction in the evolution of CRISPR immunity upon infection with 10^4^ pfu of phage (U = 0, *p* < 0.01), or a reduction in sm resistance upon infection with 10^7^ pfu of phage (U = 0, *p* < 0.01). The persistence of sensitive bacteria in QS inhibitor treatments was no longer observed when bacteria were infected with 10^9^ pfu of phage DMS3vir. Under those conditions, levels of CRISPR immunity were slightly higher compared to the control, and levels of sm resistance were slightly reduced.

### The effect of QS inhibition on phage receptor expression

We envisaged two possible reasons why in the presence of Baicalein the evolution of phage resistance might be reduced upon infection with 10^4^ or 10^7^ pfu of DMS3vir. First, it may be the case that phage amplification is less efficient in the presence of the QS inhibitor, resulting in weaker selection for resistance. Second, QS inhibition may cause cells to downregulate the phage receptors, if these are also regulated by QS [42, 43], which would cause them to become more resistant to phage infection. Indeed, the Type IV Pilus (T4P), which is the receptor of many *Pseudomonas* phage including DMS3vir [29–34, 44], is known to be regulated by QS, and manipulation of QS can therefore affect phage sensitivity [29]. To test the first hypothesis, we monitored the phage densities throughout the evolution experiment described above. This showed that in the presence of the QS inhibitor, phage amplification was higher than or equal to that observed in the control (Fig. S1). Reduced phage amplification can therefore not explain the results of this experiment.

To test the second hypothesis, that phage receptor expression is affected by QS inhibition, we measured the impact of Baicalein on *P. aeruginosa* PA14 swarming motility, a QS-regulated group behaviour which requires T4P and flagella activity [45–48]. This analysis showed that the *lasI rhlI* QS mutant was less able to swarm compared to WT cells (*p* < 0.0001) and that WT cells grown in the presence of the QS inhibitor Baicalein were no different in swarming ability to that of the QS mutants (*p* > 0.9) (Fig. S2A). As a control, we also measured swimming motility of the WT strain, which is known to be an individual behaviour driven only by flagella activity [49]. As expected, there was no significant effect of Baicalein on swimming motility in the WT and the *lasI rhlI* QS mutant strains (*p* > 0.1 in all cases) (Fig. S2B). Collectively, these data therefore confirm that QS inhibition using Baicalein inhibits a QS-controlled T4P-dependent group behaviour in PA14.

### The effect of QS inhibition on phage adsorption

**Fig. 2:**
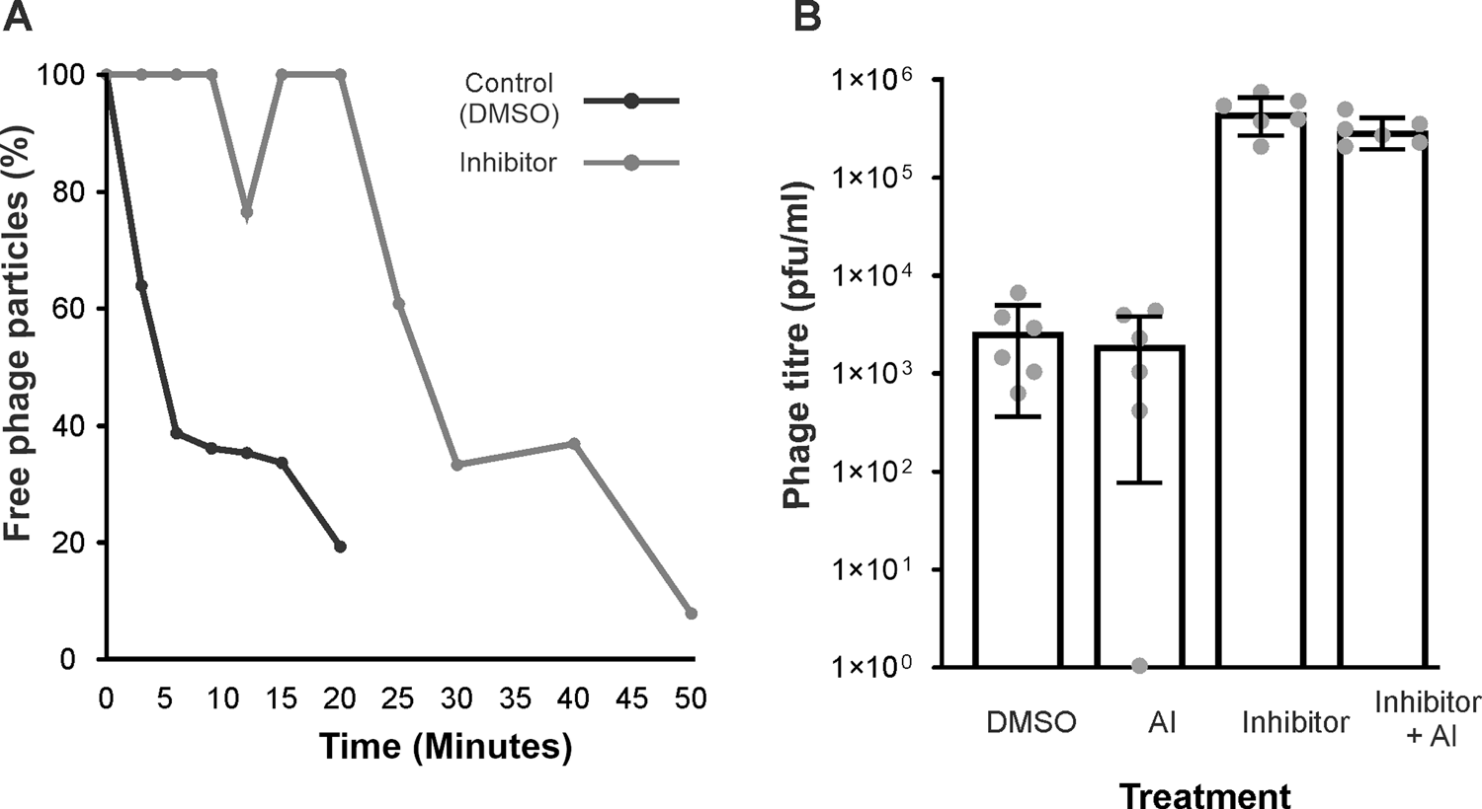
Phage are less able to infect hosts when QS is inhibited. **A** Phage adsorption over time (3–50 min) when WT *P. aeruginosa* is infected with phage when supplemented with DMSO (control) (dark grey line) or 100 µM QS inhibitor Baicalein (light grey line). Samples were taken at 3, 6, 9, 12, 15, 20, 25, 30, 40 and 50 min post infection. Lines represent mean % of free phage particles detected, *N* = 3 (**B**) Titre of phage remaining at 24hpi of a CRISPR resistant host possessing two unique spacers targeting the infecting phage (BIM2) when initially infected with 10^9^ phage. Bars represent mean titre of phage present at 24hpi, error bars indicate 95% confidence interval, points represent individual replicates, *N* = 6, Y axis is log^10^ scale.

Next, to investigate how these phenotypic changes induced by the presence of baicalein affect the infectivity of phage DMS3vir, we measured whether Baicalein affects phage adsorption. To this end, we infected WT *P. aeruginosa* PA14 cells in exponential growth phase with phage DMS3vir at a multiplicity of infection of 0.01 and measured phage titres over 50 min in the presence or absence of Baicalein (Fig. 2A). This revealed that phage took significantly longer to adsorb to host cells when the QS inhibitor was added to the media (Welch’s *t* test T = 2.548, DF = 13.78, *p* < 0.05) supporting the hypothesis that QS inhibition leads to a reduction in the rate of phage DMS3vir adsorption.

Previous work has shown that CRISPR immune bacteria cause a decline in phage densities because of their ability to continually remove phage from the population [38, 50], However only phage that adsorb to the bacterial cell and inject their genome will be targeted and removed via CRISPR-Cas activity. We therefore hypothesized that the decrease in phage adsorption observed when baicalein was present would result in higher phage titres. To test this hypothesis we infected CRISPR immune bacteria with 10^9^ pfu of DMS3vir in the presence or absence of Baicalein or synthetic autoinducers and measured phage titres after 24 h of infection (Fig. 2B). This showed that phage titres were only slightly above the threshold of detection after 24 h of infection in the control treatments. However, there was a significant decrease in the amount of phage that had been removed by CRISPR immune bacteria in the presence of the QS inhibitor (Mann–Whitney U = 0, *p* < 0.01) or the inhibitor and AIs combined (U = 0, *p* < 0.01). This is consistent with the reduction in phage adsorption in the presence of the QS inhibitors (Fig. 2A), since this leads to fewer infections and therefore a reduction in phage removal by CRISPR immune bacteria.

### The fitness effects of QS inhibition for immune, resistant and sensitive bacteria

**Fig. 3:**
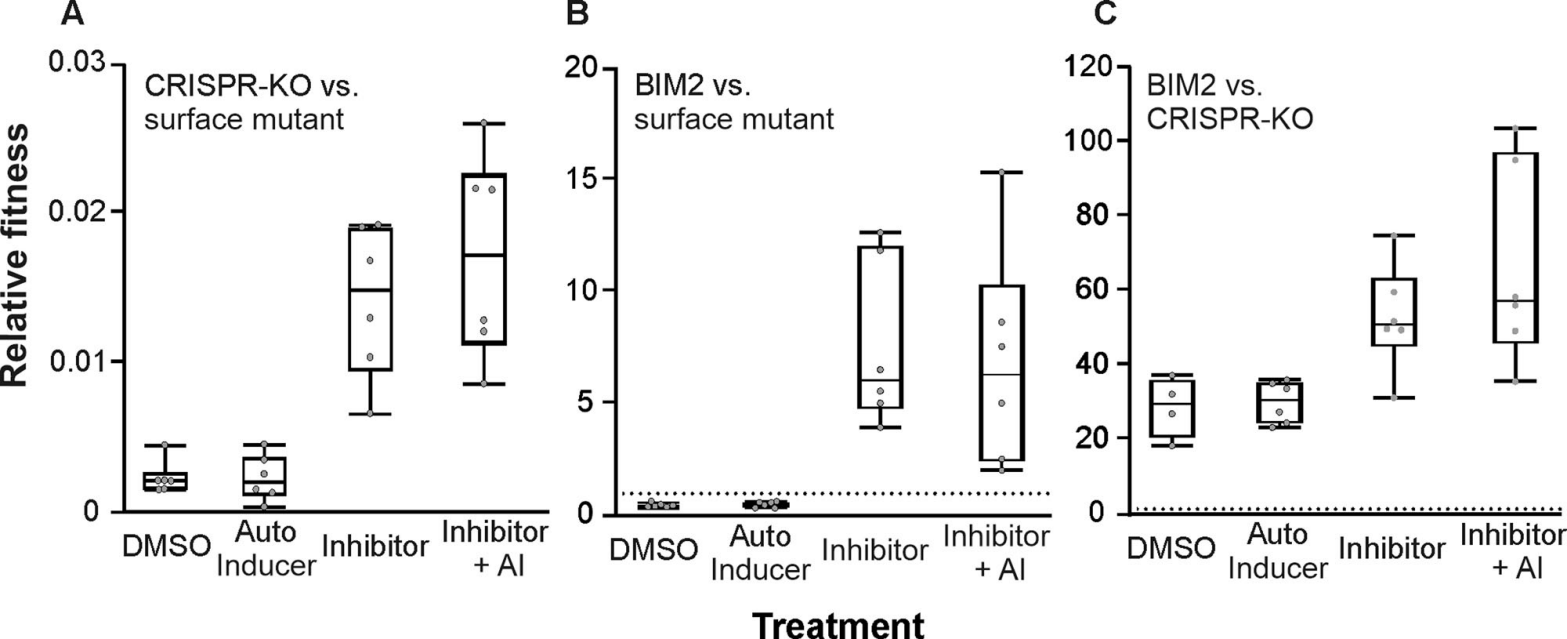
Inhibiting QS increases the fitness of *P*. *aeruginosa* in the presence of 10^6^ pfu of phage. **A** Relative fitness of sensitive CRISPR-KO strain during competition with the phage resistant surface mutant (sm). **B** Relative fitness of BIM2 during competition with a phage resistant surface mutant (sm). **C** Relative fitness of BIM2 during competition with a sensitive CRISPR-KO strain. Box plots show the median, 25th and 75th percentile, and the interquartile range. Raw values from each replicate are shown as points, *N* = 6. Dotted line in panels (**B** and **C**) indicates the point at which competing strains are equally fit (when relative fitness = 1).

Collectively, these data led us to predict that applying Baicalein may reduce the efficacy of phage-mediated killing by enhancing the intrinsic levels of phage resistance of the bacteria. To test this, we measured how Baicalein affects the fitness of phage sensitive bacteria in the presence of phage, by competing sensitive bacteria lacking a functional CRISPR-*cas* immune system (CRISPR KO strain) against a pilus deletion mutant (a *PilA* KO strain) that lacks the T4P and is fully resistant to phage DMS3vir. This showed that sensitive bacteria were always outcompeted by the pilus deletion strain in the presence of phage, as expected. However, sensitive bacteria had a significantly higher fitness when Baicalein was added to those competition experiments (Mann–Whitney U = 0, *p* < 0.005) (Fig. 3A). These data therefore confirm that Baicalein increases the resistance levels of bacteria against pilus-specific phage, hence lowering the infectivity of the phage and potentially the efficacy of phage therapy cocktails containing baicalein.

We speculated that the reduced phage adsorption in the presence of Baicalein may also explain the high levels of CRISPR immunity and low levels of sm resistance that evolve under the highest phage exposure levels (Fig. 1). CRISPR immunity is associated with an infection-induced fitness cost, whereas sm is associated with a fixed cost [14]. As a consequence, inhibition of QS and phage adsorption could lead to an enhanced fitness of CRISPR immune bacteria relative to surface mutants. Indeed, direct competition between a CRISPR immune clone (BIM2) and a bacterium with surface-based resistance (sm) revealed that the relative fitness of BIM2 increased in the presence of Baicalein (Mann–Whitney U = 0, *p* < 0.005), and was not affected by the addition of AIs (U = 17, *p* > 0.9) (Fig. 3B). Both the sm strain and BIM2 possess complete immunity against the infecting phage (no plaques observed). While the sm strain has a slightly higher fitness during phage infection in the absence of QS manipulation, the relative fitness of BIM2 is higher than that of the sm strain when QS is inhibited. The increased fitness of CRISPR immune bacteria further supports the idea that they are infected at a lower frequency when QS is inhibited.

Finally, given that CRISPR immune bacteria are less efficient in removing phage in the presence of Baicalein (Fig. 2), and sensitive bacteria will be affected by the presence of the phage more strongly than CRISPR immune bacteria, we hypothesized the outcome of direct competition between CRISPR immune bacteria and sensitive bacteria is likely to be also affected by Baicalein. Direct competition between CRISPR immune bacteria and sensitive bacteria showed that CRISPR immune bacteria outcompete sensitive bacteria in the presence of phage, as expected. Crucially, the fitness of CRISPR immune bacteria relative to sensitive bacteria increased in the presence of Baicalein (U = 2, *p* < 0.05) (Fig. 3C). By contrast, the addition of synthetic AIs had no significant impact on the relative fitness of the CRISPR immune bacteria (Mann–Whitney U = 11, *p* > 0.9), which suggests that QS systems may already be saturated under the conditions used here.

## Discussion

*P. aeruginosa* is one of the leading pathogens responsible for nosocomial infections and the most important pathogen associated with chronic airway infections in cystic fibrosis patients [51]. The rapid increase in antimicrobial resistance has led to many *P. aeruginosa* infections being very difficult to eradicate [52]. Phage therapy is therefore considered a viable alternative or complementary treatment of *P. aeruginosa* infections, and some experimental studies and clinical case studies have shown promising results [53]. However, evolution of phage resistance may limit the long-term effectiveness of phage-based therapeutics. One way to limit the evolution of phage resistance by *P. aeruginosa* is by using cocktails of different phages that use different receptors, since in this case multiple mutations in different receptor genes are needed to resist all phages [54]. When bacteria encode a CRISPR-Cas immune system, as is the case for approximately half of all sequenced *P. aeruginosa* isolates [55], this strategy may become less effective, since bacteria can in principle pick up multiple spacers that target the different phage in the cocktail [56–59]. Being able to limit the evolution of CRISPR immunity could have important clinical impact, since bacteria that remain phage sensitive or evolve CRISPR immunity are more likely to retain their virulence levels compared to bacteria that evolve surface-based resistance [11, 60, 61].

In the experiments presented here we tested the hypothesis that, because QS regulates CRISPR-*cas* expression [16, 62], chemical inhibition of QS might enhance the efficacy of phage-mediated killing of bacteria and steer evolution of phage resistance away from CRISPR immunity and towards surface-based resistance. However, in contrast to the predicted synergy between Baicalein and phage, we found that these compounds have antagonistic effects. Specifically, we found that Baicalein increased survival of sensitive bacteria and increased the fitness of sensitive and CRISPR immune bacteria relative to surface mutants. This is because QS also regulates expression of the T4P [29], which is used as a receptor by many *Pseudomonas* phage, including phage DMS3vir [44]. As a consequence, the rate of phage adsorption is reduced in the presence of Baicalein, which favours CRISPR immune bacteria over surface mutants likely because of their induced and fixed costs of resistance, respectively [14]. This shows that QS inhibition may have unanticipated effects on the evolutionary outcome of bacteria-phage interactions.

Such antagonistic effects are expected when host factors that are important for phage infection and/or replication are positively regulated by QS, whereas synergistic interactions between QS inhibitors and phage are more likely when QS downregulates the expression of phage receptors. Experimental studies with *Escherichia coli* have demonstrated QS-mediated downregulation of receptors used by its phage Lambda and Chi at high cell densities [43] and similar findings were reported for *Vibrio anguillarium*, which was more resistant to infection by its phage KVP40 at high cell densities due to QS-mediated downregulation of the phage receptor OmpK [42]. In the case of *P. aeruginosa*, the vast majority of phage characterized to date appear to either use lipopolysaccharides or the T4P as their receptor [33]. While this work shows that synergy is unlikely between pilus-specific phage and QS inhibitors, synergy between Baicalein and LPS-specific phage remains an exciting possibility that awaits experimental examination in future studies.

Manipulation of bacterial QS may also directly impact phage infection dynamics (i.e., independent of changes in host gene expression). Some temperate phage carry QS receptors [63] that can bind the autoinducers produced by the host they infect [64]. A prophage of *Vibrio cholera* that carries the QS receptor VqmA uses the autoinducers produced by the host as a cue for prophage induction when cell densities of the host are high [65]. Other temperate phage encode their own QS systems, known as Arbitrium systems, and preferentially enter the prophage state once the signal concentration becomes high, presumably because this indicates that available hosts are running out [66]. These examples highlight the importance of QS in shaping the epidemiology of phage, and manipulation of QS could conceivably alter infection dynamics and the mobility of some phage within microbial communities, which could have important implications for the bacterial population dynamics.

In summary, QS inhibitors offer exciting opportunities to manipulate the expression of genes that underpin phage resistance and clinically important traits and group behaviours such as the expression of virulence determinants [67], toxin production [68], biofilm formation [69], and swarming motility [45]. Because of the many phenotypes controlled by QS, the phenotypic, population dynamics and evolutionary consequences of QS inhibitors are difficult to predict, and experimental tests in laboratory settings and more ecologically relevant infection models will be critical to understand if and how these chemicals can be used in a clinical context and in combination with which phage.

## Supplementary information

Supplemental Information
