# Supplementary material for: The effect of Quorum sensing inhibitors on the evolution of CRISPR-based phage immunity in Pseudomonas aeruginosa
Source: ISME J. 2021 Mar 10;15(8):2465–73. doi: 10.1038/s41396-021-00946-6 (PMC8319334; doi:10.1038/s41396-021-00946-6)
Supplement: Supplementary file 1 — Supplemental Information [file 41396_2021_946_MOESM1_ESM.docx]

**Supplemental Information**

**The effect of Quorum Sensing inhibitors on the evolution of CRISPR-based phage immunity in *Pseudomonas aeruginosa***

Jenny M. Broniewski^a^, Matthew A. W. Chisnall^a^, Nina Molin Høyland-Kroghsbo^b^, Angus Buckling^a^, Edze R. Westra^a^ #

^a^Biosciences, Environment and Sustainability Institute, University of Exeter, Penryn, UK

^b^Section for Microbial Ecology and Biotechnology, Department of Plant and Environmental Sciences, University of Copenhagen, Frederiksberg C, Denmark

# Address correspondence to: [E.R.Westra@exeter.ac.uk](mailto:E.R.Westra@exeter.ac.uk)

**
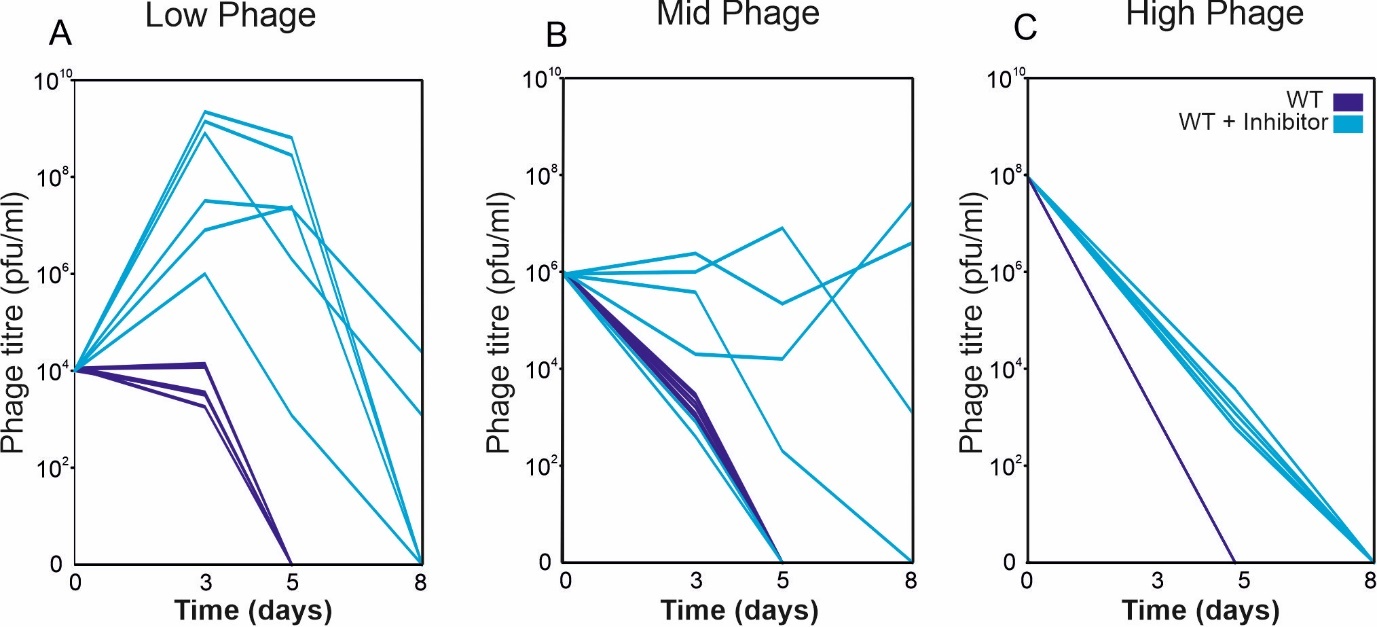
**

**Figure S1** Phage replicate more efficiently when QS is inhibited and force of infection is low. Phage densities were monitored during the evolution experiment and quantified at 3, 5 and 8dpi when initial infections were with **A)** 10^4^ pfu **B)** 10^7^ pfu **C)** 10^9^ pfu of phage DMS3vir. Phage persistence data was taken from the same experimental replicates as were analysed for the resistance mechanism graphs (Fig. 1) at the corresponding phage start density. Lines show individual replicates, N = 6.

**
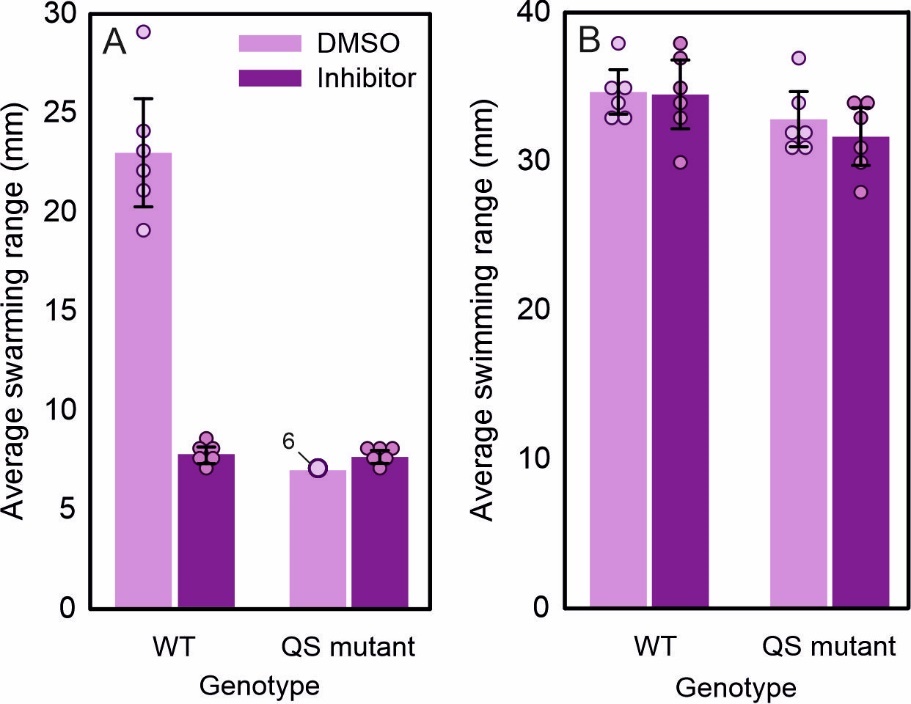
**

**Figure S2** QS inhibition reduces swarming but not swimming ability **A)** Swarming assay of WT and a *lasI rhlI* QS mutant in the presence or absence of 100 µM QS inhibitor Baicalein. **B)** Swimming motility assay of WT and a *lasI rhlI* QS mutant in the presence or absence of 100 µM QS inhibitor Baicalein. Bars represent mean growth range in mm, error bars indicate 95% confidence interval, points represent individual replicates, N = 6.
